# Evolution of the nucleus^☆^

**DOI:** 10.1016/j.ceb.2014.01.004

**Published:** 2014-06

**Authors:** Damien P Devos, Ralph Gräf, Mark C Field

**Affiliations:** 1 Centro Andaluz de Biología del Desarrollo CABD, Universidad Pablo de Olavide, Sevilla, Spain; 2 Universität Potsdam, Karl-Liebknecht-Str. 24-25, 14476 Potsdam-Golm, Germany; 3 Division of Biological Chemistry and Drug Discovery, University of Dundee, Dundee DD1 5EH, Scotland, United Kingdom

## Abstract

The nucleus represents a major evolutionary transition. As a consequence of separating translation from transcription many new functions arose, which likely contributed to the remarkable success of eukaryotic cells. Here we will consider what has recently emerged on the evolutionary histories of several key aspects of nuclear biology; the nuclear pore complex, the lamina, centrosomes and evidence for prokaryotic origins of relevant players.

## Highlights

• The nuclear pore complex is well conserved, with some regions of divergence.
• The nuclear lamina appears quite variable between major supergroups.
• Centrosomes are ancient structures, but with complex evolutionary history.
• There is evidence for prokaryotic ancestors of some nuclear components.
• Analysis of divergent organisms is essential to fully understand nuclear biology and its origins.

**Current Opinion in Cell Biology** 2014, **28**:8–15

This review comes from a themed issue on **Cell nucleus**

Edited by **Michael P Rout** and **Gary H Karpen**

For a complete overview see the Issue and the Editorial

Available online 6th February 2014

0955-0674/$ – see front matter, © 2014 The Authors. Published by Elsevier Ltd.

**http://dx.doi.org/10.1016/j.ceb.2014.01.004**

## Introduction

**Figure 1:**
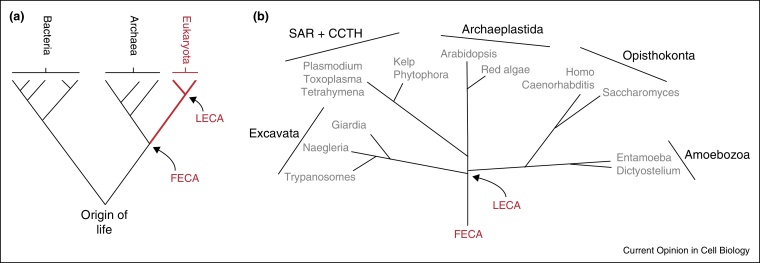
Phylogenetic tree of the current view of the topology of life and eukaryota. **(a)** Relationship between prokaryotes and eukaryotes, assuming the three-domain model, whereby the Eukaryota emerged from the Archaea. An alternate two domain model, proposes that the Eukaryotes arose as a lineage within the Archaea, but this remains unresolved [85,86]. LECA/FECA; Last/first eukaryotic common ancestor. **(b)** Eukaryotic phylogeny, based on discussions provided in [87]. Some relationships, for example within the SAR + CCTH and Excavata clades remain to be fully resolved. Examples of commonly studied and/or organisms familiar to most experimental cell biologists are provided to anchor the reader, and supergroups are indicated by bars. There is a clear emphasis within many clades in the study of pathogenic species, for obvious and fully justified reasons. SAR + CCTH; Stramenopile, Alveolata, Rhizaria + Cryptophyta, Centrohelida, Telonemia and Haptophyta.

The nucleus is enclosed by the nuclear envelope (NE) to form a container for most eukaryotic cellular DNA. Contiguous with the endoplasmic reticulum, the NE separates gene expression (transcription, mRNA maturation) from protein synthesis (translation, folding, assembly), but necessitates a channel for bidirectional trafficking (the nuclear pore complex (NPC)), a mechanism of mechanical support (lamins) and of chromosomal positioning and segregation. The NE and NPC also participate in chromosomal positioning, mitosis and transcriptional control. NE origins are linked to the ER and coated vesicles (CV) [1], probably *via* a proto-NE, that was possibly freely permeable with a sealed state arising subsequently ([2–4], discussed in [5]). Many models have been offered for nuclear origins and the events that underly the acquisition of an endomembrane system [4,6,7,8^••^] (Figure 1). Here we consider several nuclear-associated systems to provide insights into how the nucleus has evolved, together with evidence for some of the relevant prokaryotic precursors.

## The nuclear pore complex: translocator, organiser, regulator

**Figure 2:**
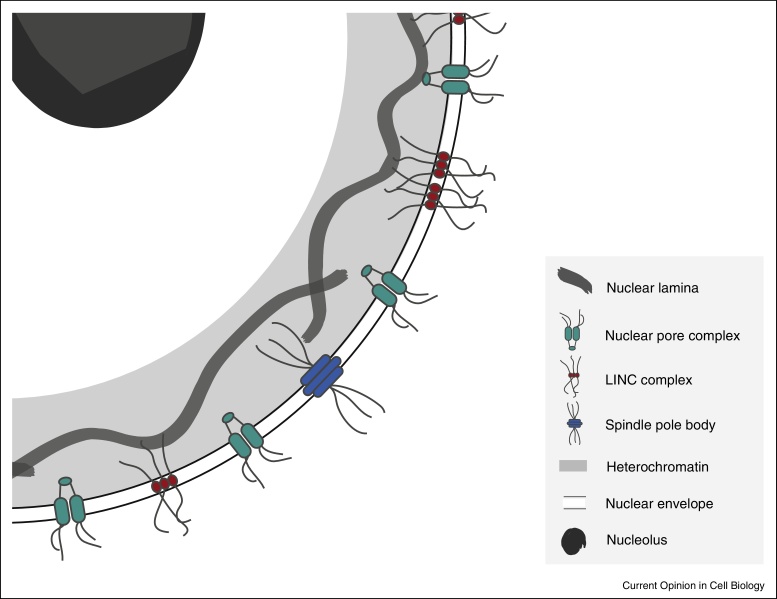
Important structures associated with the nuclear envelope. A sector of a generalised nucleus is shown, with various structures drawn as cartoons either embedded within the nuclear envelope or associated with it. Note that the structures are not drawn to an accurate scale.

Nucleocytoplasmic transport maintains a distinct composition between the cytoplasm and nucleus to facilitate functional differentiation [9,10] (Figure 2). NPCs with apparently similar morphologies are observed in the NE of many lineages, suggesting that evolutionary changes to the NPC are likely minor in terms of overall composition or architecture, and conservation of the basic mechanisms of transport across eukaryotes is clear. The NPC proteomes for yeast, mammals, trypanosomes, plants and *Tetrahymena* [11,12^••^,13–15] provide insights into NPC evolution. The NPC proteins, nucleoporins (Nups), demonstrate greatly divergent amino acid sequences but with retention of secondary structural architectures. However, *in silico* identification of Nups remains challenging and our understanding of the evolutionary histories of many individual Nups remains unclear [16,17].

**Figure 3:**
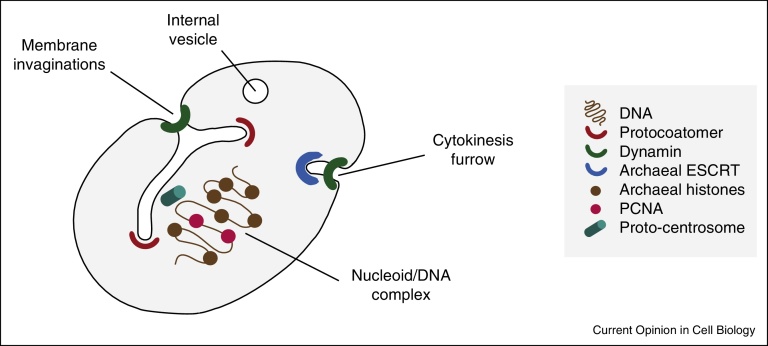
A number of features associated with prokaryotic cells that are shared with eukaryotes. Note that not all of these features are present in any one lineage. Highlighted endomembrane complexes are putative protocoatomer-like proteins that may associate with membrane in the planctomycetes, bacterial dynamin that is associated with cytokinesis and possibly other membranous structures, the partial ESCRT system found in Archaea and which plays a conserved role in cytokinesis with eukaryotes. Archaea also possess histone-like proteins and a PCNA ortholog, while it is likely that the centrosome was associated with an early membranous structure that gave rise to the nuclear envelope.

The NPC has eight spokes surrounding a central channel, and connected by the inner ring facing the channel (Nup170/Nup155 complex in yeast/metazoa), outer rings (Nup84/Nup107-160 complex in yeast/metazoa) and membrane rings (Pom152 in yeast, gp210 in metazoa) [18]. The inner and outer rings represent the structural scaffold, and most of their Nups conform to the protocoatomer architecture, that is possess β-propeller and/or α-solenoid domains, and are well conserved and structurally related to vesicle coats [1,7,19]. Further, structural similarity between some Nups and karyopherins suggest a common origin; Nup188 and Nic96 bind FG-repeats and translocate through NPCs, providing experimental evidence in support of the proposed common origin between the NPC and the soluble nuclear transport machinery [20^••^,21^••^]. This may indicate that the Kaps arose as a soluble Nup variant, or potentially *vice versa*. Some Nups, for example Seh1 and Elys are non-universal while the trypanosome Nup84 complex equivalent may possess additional subunits (S. Obado, MCF, M.P. Rout and B.T. Chait, in preparation). Most remaining Nups are conserved; Aladin for example is widely retained, but lost specifically from yeasts [5]. Clearly the major membrane-deforming/stabilising functionality of the NPC is evolutionary stable, and hence likely mechanistically similar, across the eukaryotes, consistent with a comparatively invariable morphology (Figure 3).

The membrane ring displays considerable flexibility, and the sequence divergence between yeast and animal Pom152 and gp210 is well known. Possible orthologs of gp210 and NDC1, but not Pom121, are present in *Arabidopsis* [13], but in *T. brucei* no membrane Nups have been identified to date [14]. Therefore the interface between the scaffold and NE may vary between taxa, *albeit* with unclear consequences, but may also have an association with NPC assembly [22]. FG-repeat Nups serve to provide gating functionality, and the FG/FXFG repeat, if not the precise arrangement within the Nup protein bearing the repeats, appears very widely conserved across eukaryotes. An interesting example of an exception to FG or FXFG repeat architecture comes from *Tetrahymena*, where the transcriptionally inactive micronucleus possesses Nup98 paralogs with poly-N/NIFN repeats and the transcriptionally active macronucleus, more conventional FG-repeat Nup98 [12^••^,23]. Significantly, despite variation in sequence and locations of the FG repeats in Nups, the number of FGs and sequence environment within which the FGs are embedded appear to be better conserved, implying conservation of the gating mechanism, although the precise mechanisms by which this operates remain controversial [14,16].

Both cytoplasmic fibrils and the nuclear basket exhibit complex evolutionary patterns [5], likely impacting their interactions with other cellular systems. For example, Nup358 anchors RanGAP at the cytoplasmic fibrils in metazoa but trypanosomes lack Nup358 and an alternate anchor for RanGAP is present in plants, while yeast RanGAP is solely cytoplasmic [13,16,24^••^]. Amongst the many nuclear basket connections are the transcriptional apparatus, the lamina and protein/RNA transport systems. There is evidence for conserved interactions between the NPC and TREX-2 and SAGA, important in mRNA export and transcription respectively [25] and also Nup-interactions with the spindle and checkpoint proteins [26,27^••^]. Given that TREX-2 and SAGA subunits are present in many lineages, it is again likely that this is ancient and central to NPC function. Interesting, the inner nuclear NPC components, Tpr in vertebrates and Mlps in yeasts, are orthologs and widely represented across the eukaryotes, whereas the discicristata (Euglenozoa plus Percolozoa) have no detectable Tpr/Mlp homologue, but have two nucleoporins with similar architectures and functions [5,26] (Holden *et al.*, submitted for publication). Significantly, those data suggest that LECA possessed Tpr/Mlp; the presence of analogues in early diverging trypanosomes suggests that a second mechanism was present in LECA or that this replaced an original Tpr/Mlp-based system for interacting with the nuclear interior in the discicristata, possibly as a response to changes in transcriptional mechanisms.

## Centrosomes, centrins and spindle poles

Centrosomes serve as the main microtubule-organising centres (MTOCs), and are essential for cell architecture in all organisms using microtubules for organelle positioning. Nuclear-associated bodies (NABs) or spindle pole bodies (SPBs) are centrosomal structures in association with the nucleus, and are best characterised in yeasts and *Dictyostelium* amoebae. In budding yeast the SPB consists of a stack of three plaques and is permanently inserted into the NE (Figure 2). In *Dictyostelium*, the NAB also contains a tripartite core; although attached to the NE, the *Dictyostelium* NAB is cytosolic during interphase, only entering the NE upon centrosome duplication at mitosis, similar to fission yeast [28,29]. The *Dictyostelium* NAB organises a radial microtubule cytoskeleton very similar to metazoan cells. Centrosomes of animals, yeasts and amoebozoa share a surprisingly small cohort of components: the γ-tubulin small complex (γ-TuSC; γ-tubulin, GCP2, GCP3) required for microtubule nucleation; EB1, TACC and XMAP215 for microtubule dynamics and stabilisation; centrin, Cep192/SPD2, and centrosomin (Cnn) as scaffolding proteins, kinases from the polo, aurora, NIMA and Cdk family regulating duplication and spindle organisation and the dynein motor protein [30–33]. Hence much of the diversity of centrosomal functions is likely a direct result of divergent composition in modern lineages.

The amoeboid cell state has been regarded as ancestral, and acentriolar MTOCs in fungi and amoebozoans were therefore considered to represent the primitive centrosomal form. However, comparative genomics indicates that LECA likely possessed one or two centrioles associated with a cilium, since centrioles are found in all major eukaryotic subgroups [30,34,35] and the LECA was almost definitely flagellated [8^••^]. The absence of centrioles in higher plants, fungi and most amoebozoans is therefore a secondary loss, and implies that centrosomes likely had original roles in initiating cilium formation while the centriole served as a basal body for microtubule nucleation. Indeed, ciliate centrioles act exclusively as basal bodies and their mitotic spindle poles are devoid of centrioles [36]. Centrioles may have originally exploited spindle association to ensure an equal distribution into daughter cells, rather than having an active role at the spindle [37–39], and this possibility is supported by evidence that centrioles are dispensable for spindle formation [40–42]. Despite this, these same studies found that centrioles are essential for formation of astral microtubules and cilia. The ancestral centrosome may thus have been a membrane/chromatin-associated microtubule nucleation centre with dual centromere/centrosome functions. Subsequently duplicated during eukaryotic evolution, a centrosome remained attached to the plasma membrane while a microtubule nucleation centre attached to proto-endomembranes that later differentiated into the NE [35]. This process could have generated an intranuclear microtubule nucleation centre that organised the spindle and an extra-nuclear centrosome responsible for organising pellicular and flagellum microtubules and significantly this configuration is present in the discicristata, such as Euglena and trypanosomes [43]. These scenarios suggest that the tight association of a nucleus-associated centrosome with clustered centromeres during the entire cell cycle, as in fission yeast and *Dictyostelium*, is primitive. The hypothesis that nuclear centromeres originally had dual centromere/centrosome functions is supported by observations that both structures remain closely associated with each other during the entire cell cycle, as in fission yeast or *Dictyostelium* where centromeres cluster close to the inner nuclear membrane and permanently associate with the SPB/centrosome at the cytosolic nuclear face [44–46].

Besides tubulins, centrins (of the calmodulin family of calcium-binding proteins) may be the most ancient centrosomal proteins [47], with general functions in connecting microtubular and membrane-bound structures. Centrins may have been critical to assembly of the primitive centromeric microtubule nucleation complex [35]. In *S. cerevisiae* Cdc31p (yeast centrin) is a major constituent of the assembly platform for the new SPB upon SPB duplication at the NE. There are several centrin isoforms, which in most species can be grouped into two subfamilies: human centrin-2-like and yeast Cdc31p/centrin-3-like proteins. Since members are present in both unikonts and bikonts, these subfamilies arose early [48], and losses are likely secondary events. By this model, yeasts retained only centrin-3 with its ancient, nuclear functions after loss of cilia for locomotion [35]. However, this is likely too simplistic as flies and nematodes lack centrin-3 and centrin-2 assumes the nuclear role [48]. Further, *Dictyostelium* CenA and CenB belong to neither subfamily, but both predominantly associate with the nucleus, with CenA concentrated at centromeres and CenB at nuclear internal [49,50]. While an exact function of CenA is unknown, CenB is important for nuclear architecture and centrosome nuclear attachment, the latter function being conserved with *S. cerevisiae* Cdc31p [51].

## Lamins, laminas and LINCs

The NE is subtended in most cells by a morphologically recognisable lamina, first described in amoebozoa [52–54]. The lamina in metazoan cells is comprised of lamins, a family of repetitive coiled coil ∼60–80 kDa proteins [55]. Lamins serve as organisers of heterochromatin, NPCs and multiple additional nuclear structures, reflected in the importance of laminopathies to human disease ([56], compiled in [55,57]). Lamins are targeted to the NE by C-terminal prenylation, and in mammalian cells the distinct isoforms have somewhat differing locations [58,59]. Lamins were assumed to be metazoan specific, suggesting a recent origin. It is clear this is incorrect as lamin orthologs are present in several amoebozoan species, with the best characterised being *Dictyostelium* NE81, with functions fully compatible with a *bona fide* lamin [60^••^,61], pushing the lamin origin to the origin of unikonts and perhaps even earlier. Furthermore, while there are no documented lamins within bikonts, the discicristate NUP-1 protein, and higher plant NMCP proteins assume similar locations and functions, as well as retain a predicted coiled coil architecture [55,62^••^,63^••^]. It is unknown if the LECA had a lamina of NUP-1-like, NMCP-like or lamin-like proteins, or if NUP-1/NMCP and lamins are in some manner evolutionarily related. It is formally possible that the LECA had a more complex lamina and that all but one system was subsequently lost, or that the discicristata and plants replaced a lamin-based lamina with NUP-1 or NMCP respectively.

A further group of proteins associated with the NE is SUN and KASH domain proteins [64^••^,65]. SUN proteins are present in all major eukaryotic groups, except for the discicristata [66^•^]. SUN proteins are concentrated at the inner NE and interact with KASH-family proteins at the outer NE, forming the LINC complex [64^••^,65,67]. Different KASH-family proteins manage direct or indirect connections to cytoplasmic microtubules, actin filaments, intermediate filaments and dynein, which in turn maintains the centrosome close to the nucleus through its microtubule minus end-directed motor activity. SUN proteins are linked to lamins [68], required for proper centrosome/nucleus attachment [69]. Although this linkage has been proven only for metazoa, since *Dictyostelium* NE81 is required for centrosome/nucleus attachment and interference with NE81 causes phenotypes similar to SUN1 disruptions, this likely extends to Amoebozoa [46,60^••^,70]. Thus, lamins may have co-evolved with SUN-proteins, suggesting the widespread presence of lamins, while the absence of SUN and lamins from the discicristata is compatible with the absence of lamins and substitution by NUP-1. However, as plants also have SUN/KASH and NMCP proteins but not lamins, coevolution is therefore not strictly necessary [71]. Further, a functional connection between lamins and open mitosis also can be discounted [35]. *Dictyostelium* has a partial closed mitosis, comparable to *Aspergillus* [72]; as the former has a lamin and the latter apparently does not, these features are not linked. *Dictyostelium* may have solved the problem of making the NE sufficiently flexible for karyokinesis by partial disassembly of NE81 networks, as NE81 remains associated with the NE throughout the cell cycle [60^••^]. Hence at present there remains no obvious rationale to underpin the use of a particular set of proteins to build a lamina or the functional implications of these potentially distinct systems.

## Prokaryotic origins

Given the clear conservation of many nuclear functions and structures, it is perhaps no surprise that there is growing evidence for origins of several systems and components pre-LECA, and even reaching back to prokaryotes (Table 1). Surprisingly, prokaryotic homologues of proteins with the protocoatomer architecture, that is, related to the NPC scaffold, have been detected only in bacteria belonging to the PVC superphylum [73]. PVC bacteria have a unique endomembrane system that is complex and dynamic [73–75], and it is unclear if this represents an example of convergence, lateral gene transfer or deep evolutionary relationships. Importantly, this may indicate that there is a fundamental aspect to the protocoatomer architecture that is of extreme value to membrane modeling, and further highlights that internal membranes of considerable complexity exist outside eukaryotes. Orthologs of many nuclear proteins and RNAs are present in Archaea, including PCNA, Sm-like, MCM and GINS, encompassing functions from transcription, DNA replication, mRNA processing and telomere construction [76–78]. Similarly, most archaea encode histone variants [79,80] and snoRNA genes [81], all indicating a shared cohort of nuclear genes/RNAs between Archaea and eukaryotes. With improved detection methods eukaryotic features are increasingly being identified in prokaryotes and it is becoming clear that the transition between these two major cellular forms may have been more gradual that previously suspected (Table 1).

## Summary

Many nuclear functions, including complex interactions and dynamics, are conserved across eukaryotes, and which engage massive assemblies of proteins with ancient origins. A number of notable, lineage-specific features have been described, most prominently the lamina, and the implications remain to be fully established. Furthermore, centrosomes have complex nuclear evolutionary relationships and even the strict view of an endomembrane system as a eukaryotic feature is challenged by the presence of membranous systems in prokaryotes. The emergence of additional model systems beyond the classical yeasts and animals will continue to contribute to understanding the evolution of nuclear functions and the origins of the nucleus itself.

## References and recommended reading

Papers of particular interest, published within the period of review, have been highlighted as:

• of special interest
•• of outstanding interest

## Figures and Tables

**Table 1:** Selection of genes that are represented both in prokaryotic and eukaryotic genomes. A small selection of examples is given, to illustrate that both bacteria and Archaea may share genes with eukaryotes which have important roles in the nucleus.

Protein complex	Functions in	Present in	Reference
MC proteins	Endomembrane system	Bacteria	[73]
PCNA	DNA metabolism	Archaea	[76]
Sm-like	Small nuclear ribonucleoproteins	Archaea	[77]
CMG complex	DNA replication	Archaea	[78]
snoRNA	Post-trancriptional modifications	Archaea	[81]
Dynamin	Membrane manipulation	Bacteria	[82]
ESCRT	Membrane/cell division	Archaea	[83,84]
